# Exploring the Interplay Between Radioimmunoconjugates and Fcγ Receptors in Genetically Engineered Mouse Models of Cancer

**DOI:** 10.1021/acsptsci.4c00275

**Published:** 2024-10-17

**Authors:** Cindy Rodriguez, Samantha M. Sarrett, Joni Sebastiano, Samantha Delaney, Shane A. McGlone, Meena M. Hosny, Sarah Thau, Stylianos Bournazos, Brian M. Zeglis

**Affiliations:** † Department of Chemistry, Hunter College, City University of New York, New York 10021, New York, United States; ‡ Department of Radiology, Memorial Sloan Kettering Cancer Center, New York 10021, New York, United States; § Ph.D. Program in Chemistry, Graduate Center of City University of New York, New York 10021, New York, United States; ∥ Ph.D. Program in Biochemistry, Graduate Center of City University of New York, New York 10021, New York, United States; ⊥ Laboratory of Molecular Genetics and Immunology, The Rockefeller University, 1230 York Avenue, New York 10065, New York, United States; # Department of Radiology, Weill Cornell Medical College, New York 10021, New York, United States

**Keywords:** molecular imaging, radioimmunoconjugate, positron emission tomography, PET, single photon emission computed tomography, SPECT, radioimmunotherapy, radiopharmaceutical therapy, immunotherapy, immune system, Fc receptor, Fcγ receptors, heavy chain glycans, aglycosylated antibody

## Abstract

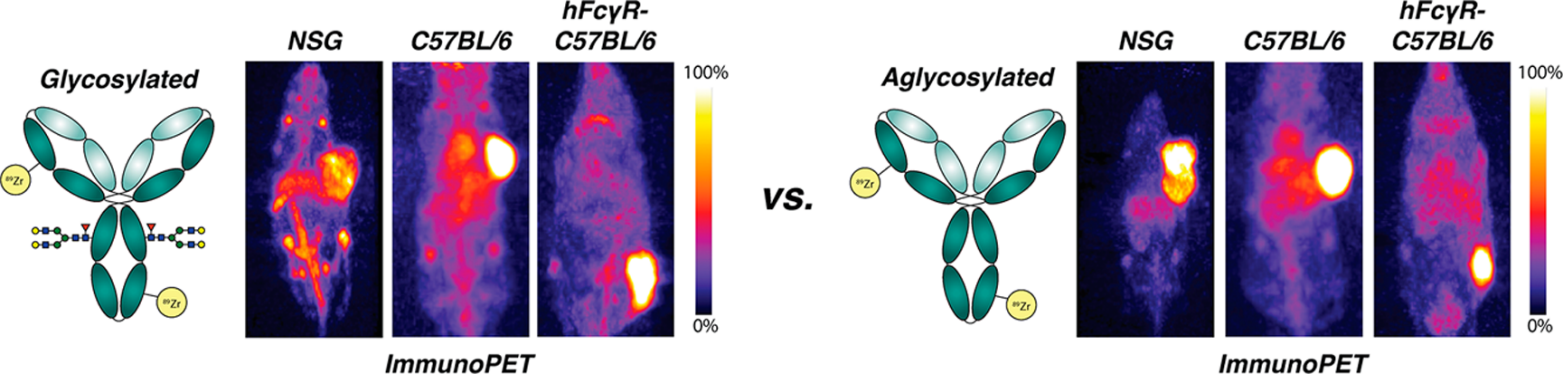

Fcγ receptors (FcγR) are responsible for many of the interactions between immunoglobulins (IgG) and immune cells. In biomedicine, this interplay is critical to the activity of several types of immunotherapeutics; however, relatively little is known about how FcγRs affect the in vivo performance of radiolabeled antibodies. A handful of recent preclinical studies suggest that binding by FcγR—and particularly FcγRI—can affect the pharmacokinetic profiles of ^89^Zr-labeled radioimmunoconjugates, but there are no extant studies in immunocompetent or genetically engineered mouse models of cancer. In the investigation at hand, we synthesized and characterized ^89^Zr-labeled probes based on wild-type and aglycosylated variants of the CA19-9-targeting antibody 5B1 and evaluated their in vivo behavior in several murine models of cancer, including immunocompetent and FcγR-humanized mice. The aglycosylated desferrioxamine (DFO)-bearing immunoconjugate DFO-^N297A^5B1 displayed identical binding to CA19-9-expressing cells compared to its wild-type analogue (DFO-5B1) but exhibited dramatically attenuated affinity for several FcγR. Positron emission tomography imaging and biodistribution studies with [^89^Zr]Zr-DFO-5B1 and [^89^Zr]Zr-DFO-^N297A^5B1 were subsequently performed in several strains of mice bearing CA19-9-expressing BxPC3 human pancreatic ductal adenocarcinoma and B16F10-FUT3 murine melanoma xenografts. Significant differences in the pharmacokinetics of the two radioimmunoconjugates were observed in tumor-bearing immunocompromised NSG mice, but these differences failed to materialize in immunocompetent C57BL/6 and FcγR-humanized C57BL/6 mice with B16F10-FUT3 xenografts. We hypothesize that these observations are related to the presence or absence of endogenous IgG. NSG mice completely lack endogenous IgG, and thus their mFcγR are free to bind radioimmunoconjugates and alter their pharmacokinetic behavior. In contrast, C57BL/6 and FcγR-humanized C57BL/6 mice both have endogenous IgG that occupy their FcγR (murine for the former and human for the latter), precluding interactions with radioimmunoconjugates. Ultimately, these data suggest that understanding the interplay between radiolabeled antibodies and FcγR is critical during the preclinical evaluation of radioimmunoconjugates.

## Introduction

Over the past quarter century, monoclonal antibodies (mAb) have become indispensable therapeutic and diagnostic tools due to their exquisite specificity and affinity for biomarkers of disease and remarkable in vivo stability. In the context of nuclear medicine, mAb have emerged as powerful vectors for the delivery of radionuclides to tumor tissue for positron emission tomography (PET), single photon emission computed tomography, and radiopharmaceutical therapy (RPT). Indeed, an ever-expanding array of radioimmunoconjugates have proven to be valuable therapeutics and diagnostics in the clinic, including [^90^Y]Y-ibritumomab tiuxetin, [^131^I]I–3F8, [^225^Ac]Ac-lintuzumab, [^89^Zr]Zr-atezolizumab, and [^89^Zr]Zr-pertuzumab.^1−5^

The critical role immunoglobulins play in the natural innate and adaptive immune responses means that the body possesses an elaborate web of proteins capable of interacting with antibodies.^6^ This presents both challenges and opportunities for those seeking to use mAb as platforms for radiopharmaceuticals. For example, the neonatal Fc receptor (FcRn) is responsible for the recycling of antibodies following endocytosis and thus contributes to the extended serum half-lives of full-length IgGs.^7^ These robust biological residence times (i.e., several days to weeks) often lead to high levels of uptake in target tissue but can also result in elevated radiation dose rates to healthy tissues, a phenomenon that has led some to pursue radioimmunoconjugates based on mAb with mutated FcRn binding sites or antibody fragments lacking Fc domains at all.^8,9^ Fcγ receptors (FcγR) represent another family of Fc-binding proteins that is responsible for many of the interactions between antibodies and immune cells, including macrophages, neutrophils, eosinophils, and dendritic cells. Several different FcγR exist—including FcγRI, FcγRIIA, FcγRIIB, FcγRIIIA, and FcγIIIB—each with its own immunoglobulin-binding properties and molecular functions, ranging from prompting phagocytosis to inducing antibody-dependent cell-mediated cytotoxicity.^6,10^

While the influence of FcRn on the pharmacokinetics of radioimmunoconjugates has long been appreciated, the impact of FcγR binding on the biodistribution of radiolabeled antibodies remains less well understood. Along these lines, FcγRI has the potential to play a particularly important role, as it boasts a far higher affinity for monomeric IgGs than either FcγRII and FcγRIII, which primarily bind immune complexes. Furthermore, FcγRI is expressed on the surface of monocytes, macrophages, and tissue-resident macrophages (e.g., Kupffer cells) in the liver and spleen and could thus contribute to the accretion of radioimmunoconjugates in these healthy tissues.^10^

Our interest in the interplay between radioimmunoconjugates and FcγR was borne out of our work on the development of site-specific bioconjugation strategies predicated on the truncation and modification of the heavy chain glycans.^11^ The heavy chain glycans are a pair of biantennary heptasaccharide chains appended to the Asn297 residues of the Fc region. It has been well established that the binding of FcγR to IgG is dependent on the heavy chain glycans, as their removal prompts the Fc region to adopt a “closed” conformation that blocks access to the FcγR binding site (but, critically, not that of FcRn).^12,13^ In our previous work, we found that ^89^Zr-labeled radioimmunoconjugates synthesized via the truncation and modification of the heavy chain glycans and fully deglycosylated probes created via traditional stochastic methods exhibited dramatically reduced binding to FcγRI compared to analogues with fully intact glycans (note: deglycosylated and aglycosylated IgG are fundamentally different: the former have had their glycans removed, while the latter never had glycans in the first place). Even more importantly, we observed that ^89^Zr-labeled variants of trastuzumab with truncated and removed glycans boasted improved in vivo performance compared to a fully glycosylated radioimmunoconjugate in NSG and huNSG mice bearing HER2-expressing human breast cancer xenografts. More specifically, the former produced higher tumoral activity concentrations and lower levels of accretion in the liver, spleen, and bones than the latter, a result that suggests that abrogating FcγRI binding can substantially improve the pharmacokinetics of a radioimmunoconjugate. Similar in vitro and in vivo results were subsequently obtained with randomly and site-specifically modified [^89^Zr]Zr-DFO-pertuzumab.^14−16^ Critically, other groups have also observed that immunoconjugates for both PET and fluorescence imaging with attenuated binding to FcγRI exhibit improved in vivo performance.^17^

While these studies have undeniably been valuable, they have all been limited to some degree by the mouse models used. In each case, the majority of experiments were performed in strains of mice (i.e., NSG and athymic) that lack competent immune systems and do not express human FcγR (hFcγR). While murine FcγR (mFcγR) *do* bind human and humanized mAb in a manner similar to their human homologues, this is still not an ideal recapitulation of the human immune milieu. In an effort to circumvent this issue, a handful of experiments were performed in humanized NSG (huNSG) mice: NSG mice sublethally irradiated 3 weeks after birth and then reconstituted with human hematopoietic stem cells to facilitate the expression of hFcγR. This mouse model (though an improvement) still has its drawbacks, however, as huNSG mice exhibit mixed FcγR expression profiles—human FcγRs for engrafted leukocytes and mouse FcγRs for several effector cells—that render the interpretation of data quite challenging.^14,15,18^

We have sought to address this limitation by employing genetically engineered FcγR-humanized mice alongside the CA19-9-targeting mAb 5B1. CA19-9—or sialyl Lewis^a^—is an established biomarker in several gastrointestinal cancers, most notable pancreatic ductal adenocarcinoma (PDAC).^19−21^ Over the past decade, 5B1 has emerged as a promising vector for the delivery of both diagnostic and therapeutic radionuclides to CA19-9-expressing tumors, yielding a wealth of preclinical and clinical data that underpins its choice as the model system for this study.^22−27^ For the investigation at hand, we created an aglycosylated mutant (N297A) of the CA19-9-targeted mAb 5B1, synthesized desferrioxamine (DFO)-bearing immunoconjugates of both the aglycosylated (DFO-^N297A^5B1) and wild-type (DFO-5B1) mAb, and interrogated their binding to a variety of FcγR. Subsequently, we labeled these probes with [^89^Zr]Zr^4+^, verified their bindings to CA19-9-expressing cancer cells, and evaluated the in vivo performance of the two radioimmunoconjugates in several murine models of cancer, including immunocompetent FcγR-humanized mice.

## Methods and Materials

### General

All reagents were purchased from Fisher Scientific (Thermo Fisher Scientific; Waltham, MA, USA) unless otherwise noted. 5B1 and ^N297A^5B1 were prepared as previously described and provided by the Bournazos Laboratory at Rockefeller University.^28^ Protein concentrations were determined via UV–vis spectroscopy using a molar absorptivity at 280 nm of 2.1 × 10^5^ M^–1^ cm^–1^ and a molecular weight of 1.5 × 10^5^ Da. All water used was ultrapure (>18.2 MΩ cm at 25 °C). *p*-SCN-Bn-DFO was purchased from Macrocyclics, Inc. (Plano, TX, USA). Matrix-assisted laser desorption/ionization (MALDI) mass spectrometry was performed by the Alberta Proteomics and Mass Spectrometry Facility (University of Alberta; Edmonton, AB, Canada). [^89^Zr]Zr^4+^ was provided by 3D Imaging (Little Rock, AR, USA).

### Instrumentation

All instruments were calibrated and maintained according to standard quality control practices and procedures. UV–vis measurements were taken on a Shimadzu BioSpecNano Microvolume UV–vis Spectrophotometer (Shimadzu Scientific Instruments; Kyoto, Japan). Radioactivity measurements were taken using a CRC-15R Dose Calibrator (Capintec, Inc.; Ramsey, NJ, USA) and Automatic Wizard^2^ gamma counter (PerkinElmer; Waltham, MA, USA).

### Synthesis of DFO-5B1 and DFO-^N297A^5B1

DFO-5B1 and DFO-^N297A^5B1 were prepared as reported previously.^15^ Briefly, 5B1 or ^N297A^5B1 (1.0 mg) in Chelex 100-treated (Bio-Rad Laboratories; Hercules, CA, USA) phosphate-buffered saline (Chelex-PBS, pH 7.4) was diluted to a final concentration of 1.0 mg/mL. The pH of the solution was adjusted to 8.8–9.0 with 0.1 M Na_2_CO_3_, 20 equiv of *p*-SCN-Bn-DFO (7.05 μL, 25 mg/mL in DMSO) were added in small aliquots, and the resulting solution was incubated on a ThermoMixer (37 °C, 500 rpm, 1 h). The immunoconjugate was purified using size exclusion chromatography (PD-10 column; GE Healthcare; Chicago, IL, USA), eluted with 2 mL of Chelex-PBS, pH 7.4, and concentrated using 2 mL Amicon Ultra centrifugal filters with a 50 kDa molecular weight cutoff (MWCO; MilliporeSigma).

### Determining the Chelator-to-Antibody Ratio (CAR)

The chelator-to-antibody (CAR) of ∼1.4 DFO-chelator per antibody for DFO-5B1 and DFO-^N297A^5B1 was determined in triplicate using an Ultraflex MALDI tandem time-of-flight (ToF) mass spectrometer (Bruker Daltonics GmbH; Bremen, Germany). Each sample (1 mg/mL in water) was mixed with sinapinic acid (10 mg/mL in 50% acetonitrile/water and 0.1% trifluoroacetic acid) at 1:1, with 1 μL of the sample/matrix solution spotted onto a stainless-steel target plate and air-dried. Ions were analyzed in positive mode and externally calibrated with a bovine serum albumin standard. The difference between the mass of each DFO-bearing antibody and its unmodified counterpart was calculated, with the CAR determined via division by the mass of the chelator.

### SDS-PAGE

5B1, ^N297A^5B1, DFO-5B1, and DFO-^N297A^5B1 were characterized under reducing conditions by sodium dodecyl sulfate-polyacrylamide gel electrophoresis (SDS-PAGE) as previously reported.^15^

### ELISA

Recombinant human FcγRI/CD64, FcRn, FcγRIIA, FcγRIIB, FcγRIIIA, or FcγRIIIB (SinoBiological) was diluted to 2 μg/mL in sterile PBS, and 100 μL/well was coated onto an ELISA plate (Plates-Nunc MaxiSorp flat-bottom 96 well plate, Fisher Scientific) overnight at 4 °C. The following morning, the wells were washed 3× with PBS + Tween (0.05%), and blocking was performed with PBS containing 10% FCS. For the assays assessing monomeric interactions (e.g., between mAbs and FcγRI or FcRn), the immunoconjugates were diluted in blocking buffer—i.e. a serial dilution of 1–0.0005 μg/mL for FcγRI and 1 μg/mL for FcRn—and 100 μL was incubated in each well for 2 h at RT. For the assays with FcRn, all buffers were adjusted to pH 6 with 1 M HCl. For the assays interrogating interactions with immune complexes (i.e., those of FcγRIIA/B and FcγRIIIA/B), the immunoconjugates were diluted in blocking buffer (10 μg/mL) and heated at 60 °C for 30 min to facilitate the formation of soluble immune complexes. Subsequently, 100 μL of this solution was incubated in each well for 2 h at RT. The immunoconjugates were detected using 1:5000 HRP-labeled goat anti-human secondary IgG (JacksonImmunoResearch Laboratories, West Grove, PA, USA). After a final wash step, TMB substrate was used to develop the bound HRP secondary antibody, and the color reaction was stopped with 2 N H_2_SO_4_. Optical densities at 450 nm were determined using a SpectraMax i3 plate reader (Molecular Devices). Binding data was collected in triplicate, averaged, and plotted.

### Radiolabeling

DFO-5B1 or DFO-^N297A^5B1 was radiolabeled with [^89^Zr]Zr^4+^ according to standard published protocols.^29^ Briefly, each immunoconjugate (0.5 mg) was diluted in Chelex-treated PBS to a final concentration of 0.5 mg/mL. [^89^Zr]Zr^4+^ [92.5 MBq (2.5 mCi)] in 1.0 M oxalic acid was then diluted with Chelex-treated PBS, and the solution pH was adjusted to 7.0–7.5 with 1.0 M Na_2_CO_3_ (final volume 100 μL). After the bubbling of CO_2(g)_ stopped, the solution of [^89^Zr]Zr^4+^ was added to the solution of mAb, mixed thoroughly, and incubated on a ThermoMixer for 15 min at 500 rpm and 37 °C. The progress of the reaction was monitored via radio-iTLC with an eluent of 50 mM EDTA, pH 5.0, an AR-2000 Radio-TLC plate reader, and Winscan Radio-TLC software (Bioscan, Inc.; Washington, DC, USA). Once the reaction reached completion, free [^89^Zr]Zr^4+^ was removed via size exclusion chromatography. The radiochemical purity of the final radiolabeled construct was assayed using radio-iTLC with an eluent of 50 mM EDTA, pH 5.0.

### In Vitro Stability Assays

Following radiolabeling with [^89^Zr]Zr^4+^, 500 μCi of [^89^Zr]Zr-DFO-5B1 or [^89^Zr]Zr-DFO-^N297A^5B1 were incubated in human serum at 37 °C for 120 h. The stability of the radioimmunoconjugates to demetalation was measured every day via radio-iTLC and every other day via SEC-HPLC.

### Cell Culture

The human pancreatic cancer cell line BxPC3 and the murine melanoma cell line B16F10 were purchased from the American Type Culture Collection in 2020 and periodically authenticated by the Specimen Processing/Research Cell Bank Shared Resource using Short Tandem Repeat Combined DNA Index System typing. B16F10-FUT3 cells—i.e. B16F10 cells that had been transduced with fucosyltransferase III (FUT3) to express human CA19-9—were prepared by and acquired from the Bournazos Lab in Rockefeller University according to published protocols. Both B16F10 cell lines were maintained in Dulbecco’s Modified Eagle Medium supplemented with 10% heat-inactivated fetal calf serum, 100 units/mL penicillin, and 100 units/mL streptomycin in an incubator at 37 °C and 5% CO_2(g)._ The BxPC3 cell line was maintained in RPMI-1640 Medium supplemented with 10% heat-inactivated fetal calf serum, 100 units/mL penicillin, and 100 units/mL streptomycin in an incubator at 37 °C and 5% CO_2(g)._ The cells were passaged upon reaching 80% confluency. Aggregates that may have formed in suspension were dissociated by incubating the cells with Gibco TrypLE Express Enzyme (1×) with phenol red (ThermoFisher) for 5 min between each passage.

### Immunoreactivity Measurements

Immunoreactivity measurements were performed according to published protocols. Briefly, 1 ng of purified [^89^Zr]Zr-DFO-5B1 or [^89^Zr]Zr-DFO-^N297A^5B1 was incubated with 20 × 10^6^ BxPC3, B16F10, or B16F10-FUT3 cells in 200 μL of cold media for 1 h at 4 °C (*n* = 3 for each radioimmunoconjugate). The cells were then spun down to a pellet at 650 rcf for 3 min, and the supernatant was collected. The cell pellets were washed 2× with 500 μL of cold cell media, and both washes were collected. The amount of radioactivity in the cell pellets, supernatant, and washes were measured using an Automatic Wizard^2^ γ-counter (PerkinElmer) calibrated for ^89^Zr. The percent immunoreactivity was calculated using the ratio of the counts present in the cell pellet against the total counts in the cell pellet, supernatant, the first wash, and the second wash.

### Mouse Models and Animal Care

Five to eight-week-old female NSG mice (#005557) and C57BL/6 mice (#000664) were obtained from The Jackson Laboratory. Genetically engineered Fc-humanized C57BL/6 mice were developed by the Laboratory of Molecular Genetics at Rockefeller University as previously described and were obtained at 7 months-old.^30^ All mice were allowed to acclimatize approximately 1 week prior to inoculation. The animals were housed in ventilated cages and given food and water *ad libitum*. All animal work was approved by the IACUCs of Hunter College and Weill Cornell Medical College.

### Subcutaneous Xenografts

Subcutaneous xenografts were used for all in vivo studies. To this end, mice were anesthetized by inhalation of 2% isoflurane/oxygen gas mixture (Baxter Healthcare; Deerfield, IL, USA). The injection site was sanitized with an ethanol wipe, and 5 × 10^6^ BxPC3, 1 × 10^6^ B16F10, or 1 × 10^6^ B16F10-FUT3 cells (100 μL) in 1:1 Matrigel/PBS (BxPC3) or sterile PBS (B16F10) were injected subcutaneously in the right shoulder. The tumors reached an acceptable size for experimentation (∼100 mm^3^) after approximately 2 weeks (BxPC3) or 1 week (B16F10).

### PET Imaging

PET images of the mice were acquired using either a microPET Focus 120 (Siemens Medical Solutions) or an Inveon PET small animal imaging system (Siemens Medical Solutions; Malvern, PA, USA). Following the intravenous tail vein administration of either [^89^Zr]Zr-DFO-5B1 or [^89^Zr]Zr-DFO-^N297A^5B1 [3.7–3.9 MBq (100–105 μCi), 20–21 μg in 100 μL PBS], the mice (*n* = 4 per group) underwent 10–30 min static scans every 24 h until 120 h post-injection. The counting rates in the reconstructed images were converted to activity concentrations (percentage injected dose per gram of tissue [%ID/g]) using a system calibration factor derived from the imaging of a mouse-sized water-equivalent phantom containing ^89^Zr. Image reconstruction was performed via 3-dimensional ordered subsets expectation maximization (3D-OSEM). The data were processed using ASIPro VM software (Concorde Microsystems).

### Biodistribution Studies

Following the final time point of the PET imaging studies (i.e., 120 h post-injection), the mice were euthanized via CO_2*(g)*_ asphyxiation followed by cervical dislocation. Selected organs were collected, rinsed in water, dried, weighed, and quantified using an Automatic Wizard^2^ γ-counter (PerkinElmer) calibrated for ^89^Zr. The counts/min in each tissue were corrected for background and decay to the start of the activity measurement. The %ID/g for each sample was calculated by normalization to the total injected activity.

### Statistical Analysis

The statistical analyses were performed using GraphPad Prism 7.0 software. All data are expressed as mean ± SD. When applicable, statistical differences were analyzed by an unpaired, two-tailed Student’s *t*-test (with a Welch’s correction when mentioned) and the one-way ANOVA for the comparison of more than two groups. Differences at the 95% confidence level (*P* < 0.05) were considered statistically significant.

## Results

### Bioconjugation and Radiopharmaceutical Chemistry

The CA19-9-targeting mAb 5B1 was chosen as the model system for this investigation because it represents a well-studied (and clinically validated) platform for immunoPET.^24,31^ Accordingly, zirconium-89 (^89^Zr) was selected as the radionuclide because its physical half-life (*t*_1/2_ = 3.3 d) aligns well with the biological residence time of mAb, and desferrioxamine (DFO) was used because it is currently the clinical “gold-standard” chelator for [^89^Zr]Zr^4+^.^5,29^

**Figure 1:**
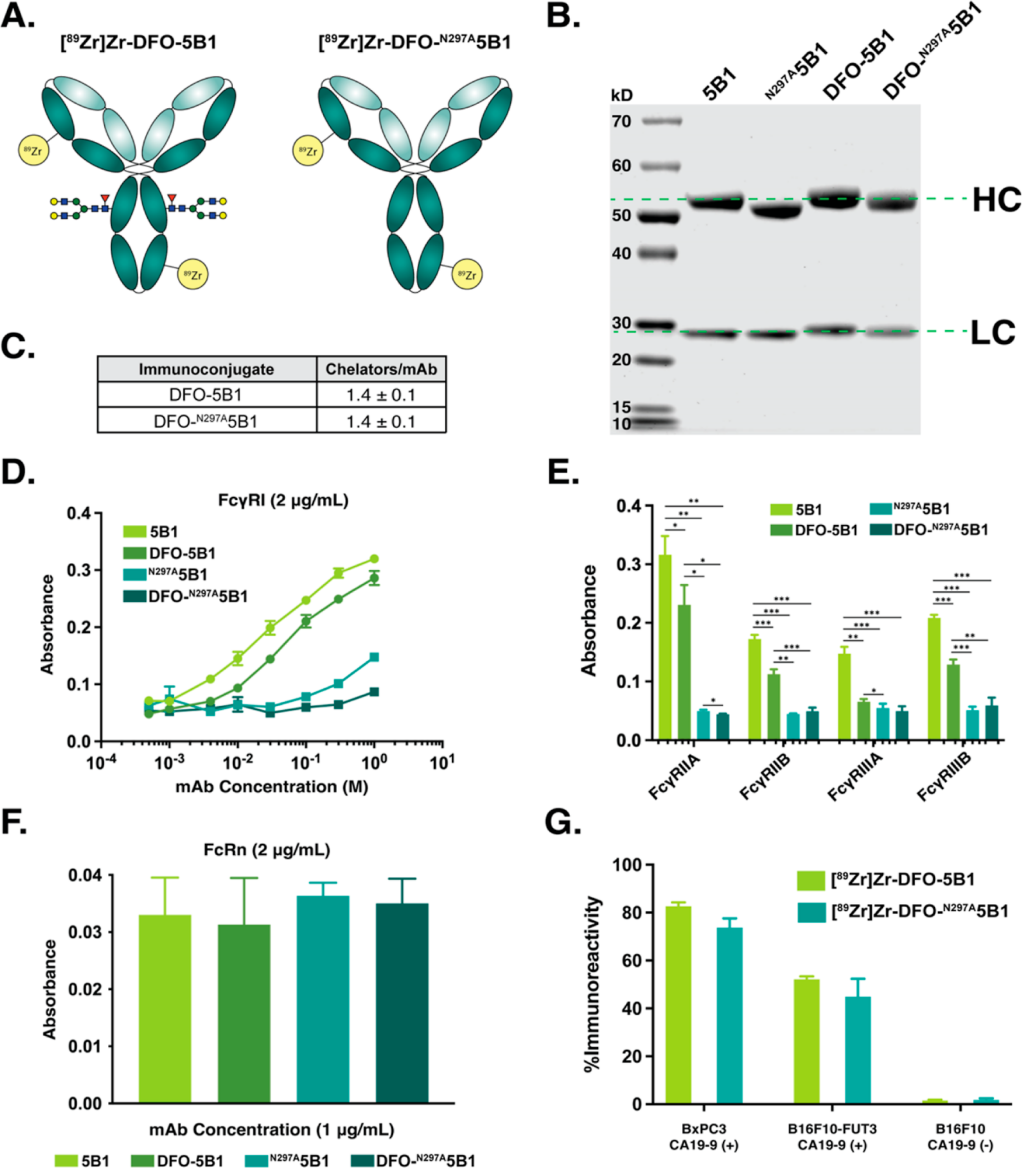
(A) Schematics of [^89^Zr]Zr-DFO-5B1 and [^89^Zr]Zr-DFO-^N297A^5B1. (B) Protein-stained SDS-PAGE of the immunoconjugates and their parent antibodies. (C) Chelator-to-antibody ratio values as determined by MALDI-ToF. ELISA assays exploring the binding of 5B1, DFO-5B1, ^N297A^5B1, and DFO-^N297A^5B1 to hFcγRI (D), hFcγRIIA/B and hFcγRIIIA/B (E), and FcRn (F). (G) Cell-based immunoreactivity data for [^89^Zr]Zr-DFO-5B1 and [^89^Zr]Zr-DFO-^N297A^5B1 obtained using CA19-9-expressing BxPC3 PDAC and B16F10-FUT3 murine melanoma cells as well as a B16F10 cell line that does not express CA19-9 (*n* = 3). *p < 0.05; **p < 0.01; ***p < 0.001.

The first step in the investigation was the synthesis and characterization of the DFO-bearing immunoconjugates (Figure 1A). To this end, a variant of 5B1 bearing N297A mutations that preclude glycosylation (^N297A^5B1) was prepared, and the absence of the heavy chain glycans was confirmed via SDS-PAGE and MALDI-ToF spectrometry (Figures 1B,C, S1, and S2). Subsequently, both wildtype 5B1 and ^N297A^5B1 were stochastically modified with *p*-NCS-Bn-DFO as previously reported, producing a pair of immunoconjugates—DFO-5B1 and DFO-^N297A^5B1—with CARs of ∼1.4 DFO/mAb as determined by MALDI-ToF. The radiolabeling of the DFO-bearing immunoconjugates with [^89^Zr]Zr^4+^ was performed according to published protocols.^29^ The radioimmunoconjugates—[^89^Zr]Zr-DFO-5B1 and [^89^Zr]Zr-DFO-^N297A^5B1—were synthesized in >99% radiochemical conversion and isolated in >99% purity and specific activities of ∼5 mCi/mg (185 MBq/mg) (Figure S3). Subsequently radio-iTLC and SE-HPLC assays revealed that both probes remained >90% stable to demetalation and aggregation after incubation in human serum for 120 h at 37 °C.

### In Vitro Characterization

The binding of FcRn and several FcγRs to the two DFO-bearing immunoconjugates and their parent mAbs was interrogated via ELISA. These assays clearly revealed that the absence of the glycans reduces the binding of all FcγRs to both the parent mAbs and the immunoconjugates. Interestingly, this trend was observed for the receptors that bind monomeric mAb (i.e., FcγRI) *and* those binding immunocomplexes (i.e., FcγRIIA, FcγRIIIB, FcγRIIIA, and FcγRIIIB) (Figure 1D,E). The presence or absence of the DFO chelator has little impact on the affinity of the FcγRs, reinforcing that glycosylation state is the key factor. Finally, all four immunoglobulins demonstrated equivalent binding to FcRn, not a surprising result given that the receptor’s binding to mAb has been well reported to be independent of glycosylation (Figure 1F). Subsequently, the ability of [^89^Zr]Zr-DFO-5B1 or [^89^Zr]Zr-DFO-^N297A^5B1 to bind CA19-9 was explored via immunoreactivity assays with CA19-9 positive BxPC3 human PDAC cells as well as isogenic CA19-9-negative (B16F10) and CA19-9-positive (B16F10-FUT3) murine melanoma cells. The tests revealed that the glycosylated and aglycosylated radioimmunoconjugates exhibited immunoreactivities of 83 ± 2% and 74 ± 4% with the BxPC3 cells, 52 ± 1% and 45 ± 8% with the B16F10-FUT3 cells, and 1.5 ± 0.3% and 1.8 ± 0.7% B16F10 cells (Figure 1G). These data reinforce the specificity of the radioimmunoconjugates and confirm that the glycosylation state of the probe does not affect its ability to bind its antigen. These numbers are admittedly lower than typical immunoreactivity values for ^89^Zr-labeled mAb but are consistent with results previously obtained for radioimmunoconjugates based on 5B1, a phenomenon that likely stems from the shed nature of its carbohydrate antigen.

### In Vivo Evaluation

**Figure 2:**
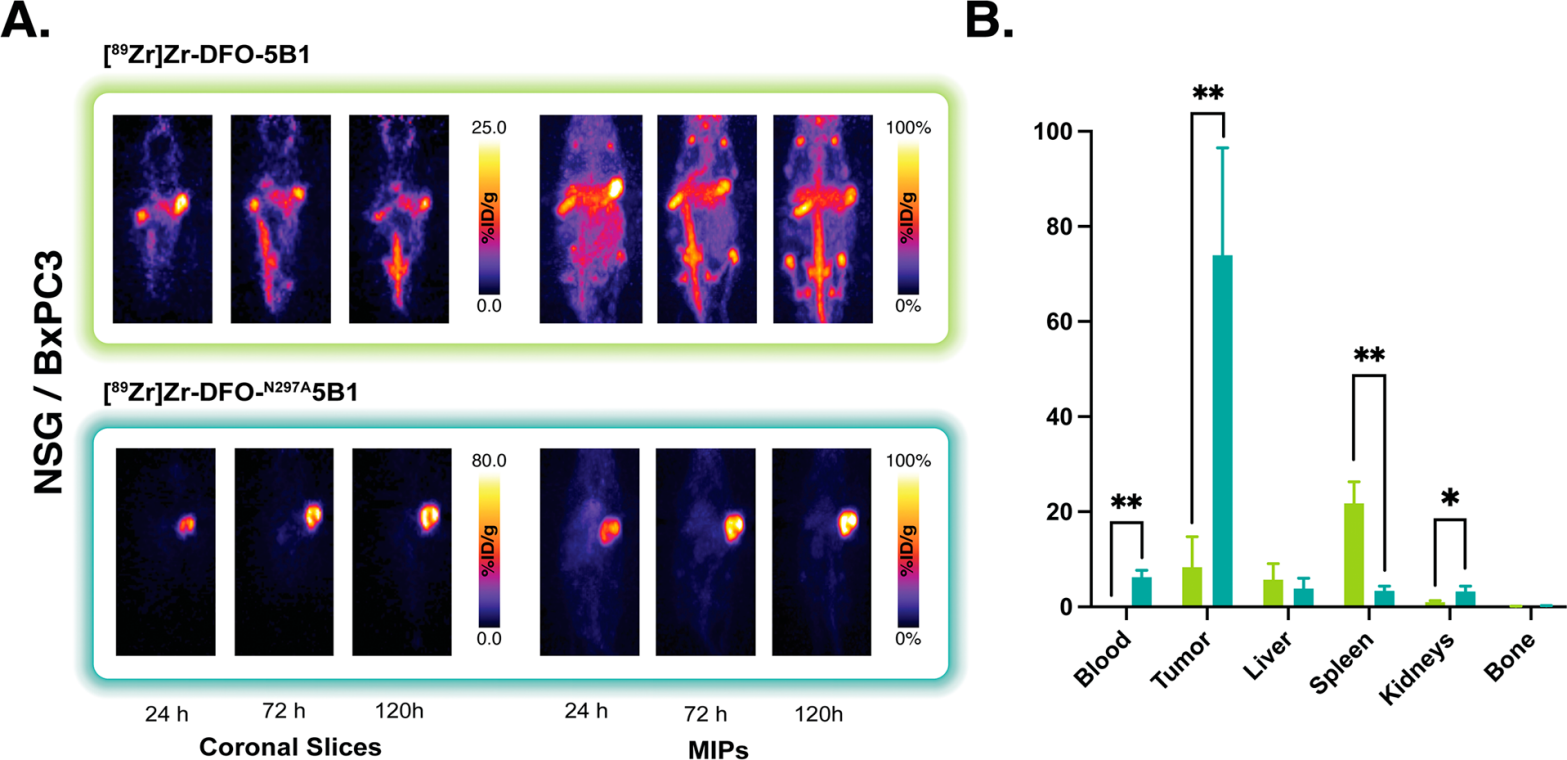
(A) Representative coronal PET images and maximum intensity projections (MIP) collected 24, 72, and 120 h after the intravenous administration of [^89^Zr]Zr-DFO-5B1 or [^89^Zr]Zr-DFO-^N297A^5B1 [3.7–3.9 MBq (100–105 μCi); 20–21 μg; in 100 μL PBS] to NSG mice bearing subcutaneous CA19-9-expressing BxPC3 human PDAC xenografts (*n* = 4). (B) Biodistribution data collected from each cohort of mice 120 h after the administration of the two radioimmunoconjugates. *p < 0.05; **p < 0.01.

**Figure 3:**
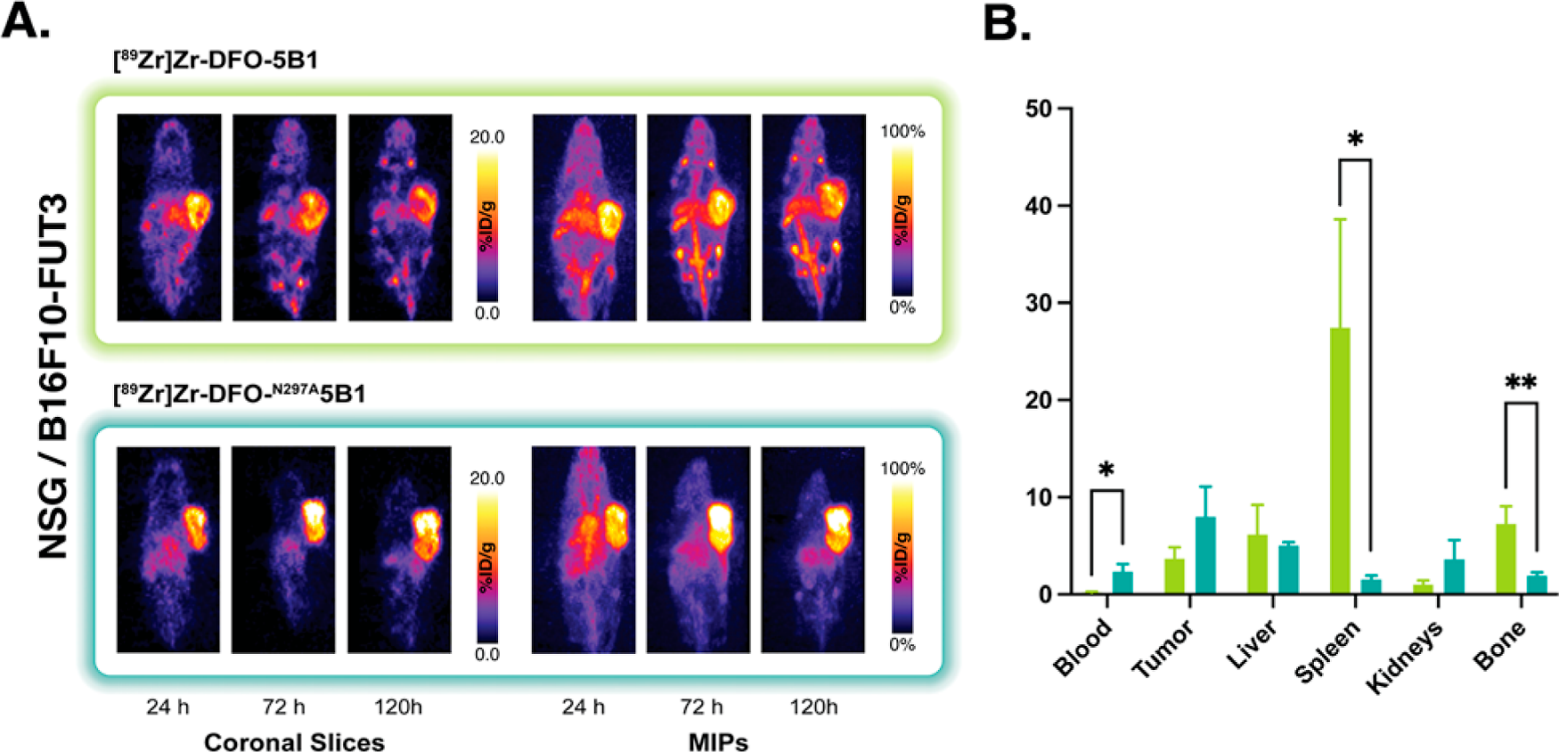
(A) Representative coronal PET images and MIP collected 24, 72, and 120 h after the intravenous administration of [^89^Zr]Zr-DFO-5B1 or [^89^Zr]Zr-DFO-^N297A^5B1 [3.7–3.9 MBq (100–105 μCi); 20–21 μg; in 100 μL PBS] to NSG mice bearing subcutaneous B16F10-FUT3 murine melanoma xenografts (*n* = 4). (B) Biodistribution data collected from each cohort of mice 120 h after the administration of the two radioimmunoconjugates. *p < 0.05; **p < 0.01.

Without question, the most important step in this investigation was the in vivo evaluation of the glycosylated and aglycosylated radioimmunoconjugates in several murine models of CA19-9-expressing cancer. Our first set of experiments was performed in highly immunodeficient NOD scid gamma (NSG; NOD.CgPrkdcscidIl2rgtm1Wjl/SzJ) mice that express mFcγR but lack T-cells, B-cells, and functional NK cells. The mice (*n* = 4 per group) were inoculated with subcutaneous BxPC3 human PDAC xenografts and subsequently administered 100 μCi (3.7 MBq, 20 μg) of either [^89^Zr]Zr-DFO-5B1 or [^89^Zr]Zr-DFO-^N297A^5B1 via the lateral tail vein. PET images were then acquired at 24, 72, and 120 h post-injection, and biodistribution data were collected after the final imaging scan (Figure 2). While both radioimmunoconjugates clearly visualized the tumor tissue, stark differences between the two sets of images were observed at 24 h and continued to the end of the experiment. More specifically, [^89^Zr]Zr-DFO-^N297A^5B1 produced higher activity concentrations in tumor tissue and lower accretion levels in the liver, spleen, and bone than [^89^Zr]Zr-DFO-5B1. These data were corroborated by biodistribution data (Figure 3). [^89^Zr]Zr-DFO-^N297A^5B1 boasted a much higher activity concentration in the tumor at 120 h p.i. than [^89^Zr]Zr-DFO-5B1: 74.0 ± 22.6 vs 8.3 ± 6.4 %ID/g. Conversely, the glycosylated radioimmunoconjugate produced higher uptake in the spleen (21.8 ± 4.5 %ID/g) and bone (7.5 ± 1.8 %ID/g) compared to the aglycosylated variant (3.4 ± 1.0 and 2.5 ± 0.4 %ID/g, respectively) at the same time point.

The next set of experiments was performed in NSG mice bearing subcutaneous xenografts created using murine melanoma cells (B16F10-FUT3) that had been transfected to express CA19-9, as these cells could be used for experiments in both immunocompromised and immunocompetent mice (vide infra). Again, the tumor bearing mice were administered [^89^Zr]Zr-DFO-5B1 or [^89^Zr]Zr-DFO-^N297A^5B1 (100 μCi; 3.7 MBq; 20 μg) via the lateral tail vein; PET images were collected at 24, 72, and 120 h post-injection; and biodistribution data were acquired after the final scans were taken. The biodistribution profiles of the two radioimmunoconjugates showed trends similar to those observed in the BxPC3-bearing NSG mice (Figure 3). [^89^Zr]Zr-DFO-^N297A^5B1 produced higher activity concentrations in tumor tissue (8.0 ± 3.1 %ID/g) compared to [^89^Zr]Zr-DFO-5B1 (3.7 ± 1.2 %ID/g). In addition, the aglycosylated radioimmunoconjugate produced far lower uptake values in the spleen (1.6 ± 0.4 %ID/g) and bone (1.9 ± 0.3 %ID/g) compared to its fully glycosylated cousin (27.5 ± 11.1 and 7.3 ± 1.8 %ID/g, respectively).

**Figure 4:**
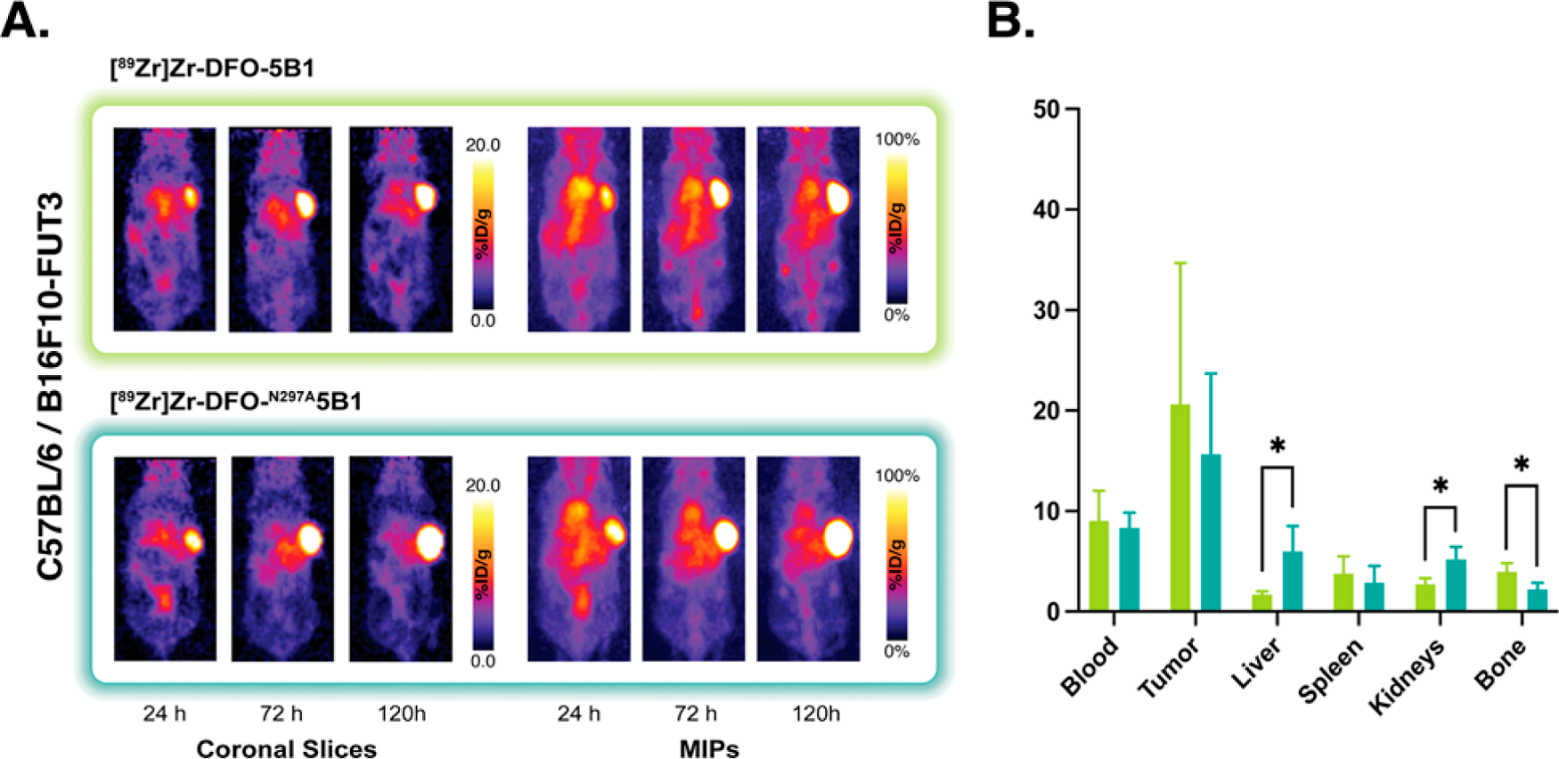
(A) Representative coronal PET images and MIP collected 24, 72, and 120 h after the intravenous administration of [^89^Zr]Zr-DFO-5B1 or [^89^Zr]Zr-DFO-^N297A^5B1 [3.7–3.9 MBq (100–105 μCi); 20–21 μg; in 100 μL PBS] to C57BL/6 mice bearing subcutaneous B16F10-FUT3 murine melanoma xenografts (*n* = 4). (B) Biodistribution data collected from each cohort of mice 120 h after the administration of the two radioimmunoconjugates. *p < 0.05; **p < 0.01.

In search of more realistic recapitulations of the interactions between radioimmunoconjugates and the immune system, we next turned to immunocompetent mouse models. We first performed PET and biodistribution studies with both radioimmunoconjugates in fully immunocompetent C57BL/6 mice bearing CA19-9-expressing B16F10-FUT3 melanoma xenografts. In this model system, the pharmacokinetic differences that we had previously observed in NSG mice failed to materialize. Both [^89^Zr]Zr-DFO-^N297A^5B1 and [^89^Zr]Zr-DFO-5B1 visualized tumor tissue very effectively, with the xenograft the most pronounced feature in the PET images by 72 and 120 h post-injection (Figure 4). Furthermore, biodistribution data revealed that at 120 h, there were no significant differences between the uptake of the aglycosylated and glycosylated radioimmunoconjugates in the tumor (15.7 ± 8.0 vs 20.7 ± 14.0 %ID/g, respectively), spleen (2.9 ± 1.6 vs 3.8 ± 1.7 %ID/g), or bone (2.2 ± 0.7 vs 3.9 ± 0.8 %ID/g) (Figure 4).

**Figure 5:**
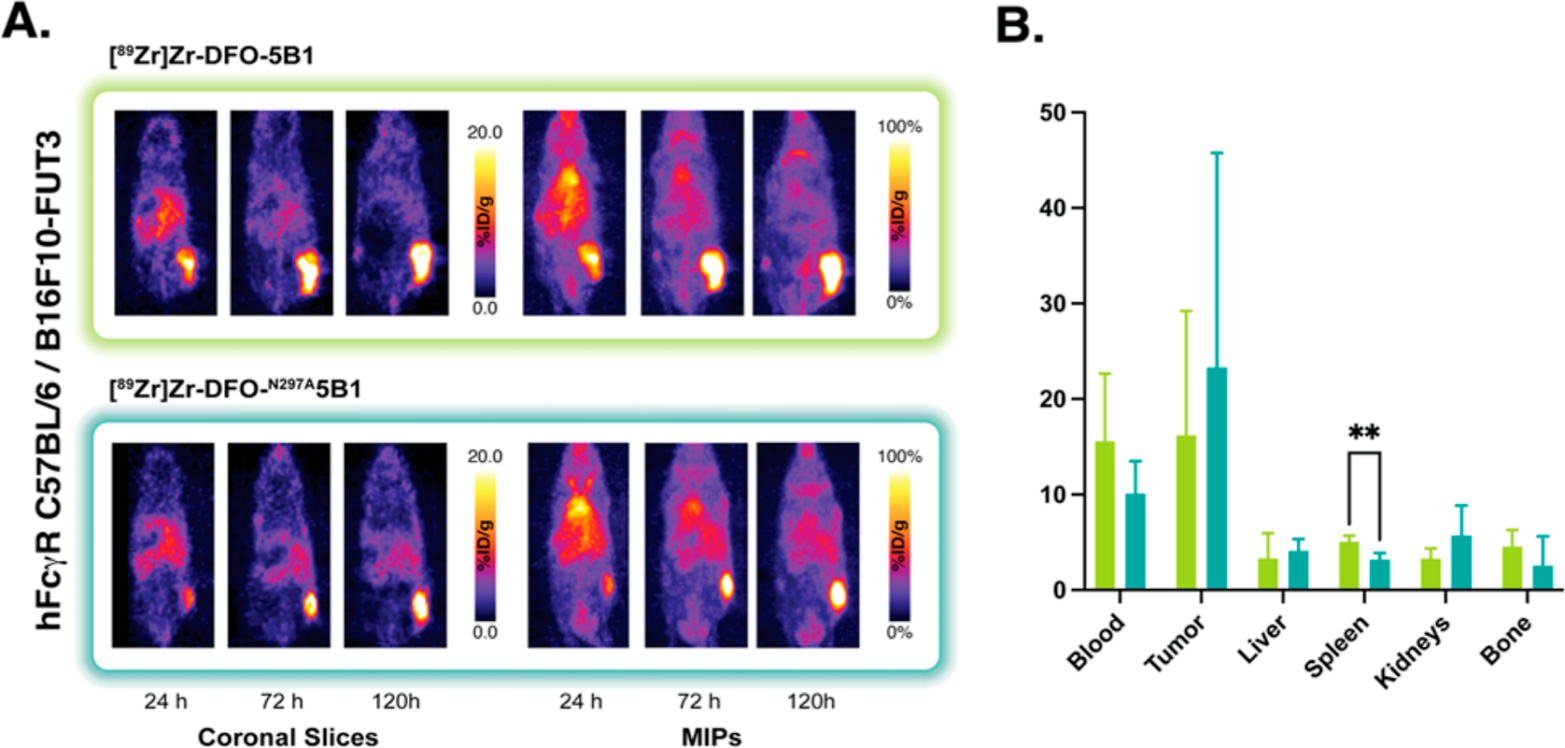
(A) Representative coronal PET images and MIP collected 24, 72, and 120 h after the intravenous administration of [^89^Zr]Zr-DFO-5B1 or [^89^Zr]Zr-DFO-^N297A^5B1 [3.7–3.9 MBq (100–105 μCi); 20–21 μg; in 100 μL PBS] to FcγR-humanized C57BL/6 mice bearing subcutaneous B16F10-FUT3 murine melanoma xenografts (*n* = 4). (B) Biodistribution data collected from each cohort of mice 120 h after the administration of the two radioimmunoconjugates. *p < 0.05; **p < 0.01.

While C57BL/6 mice certainly represent an improvement over NSG mice, the former still express mFcγRs, receptors that can bind human IgGs but with affinities well below that of hFcγRs. To circumvent this issue, we turned to a strain of C57BL/6 mice genetically engineered to express a full complement of hFcγR. Unlike huNSG mice, these FcγR-humanized C57BL/6 mice are immunocompetent and exclusively express hFcγR across all types of leukocytes (i.e., neutrophils, monocytes, macrophages, etc.). As in the previous experiments, these mice were inoculated with subcutaneous B16F10-FUT3 xenografts and intravenously injected with either [^89^Zr]Zr-DFO-^N297A^5B1 and [^89^Zr]Zr-DFO-5B1, and the in vivo performance of the two radioimmunoconjugates was interrogated via PET (24, 72, and 120 h p.i.) and biodistribution (120 h p.i.). The PET images revealed that the two radioimmunoconjugates exhibit strikingly similar behavior (Figure 5). Both clearly delineate tumor tissue as early as 24 h p.i. and largely clear from healthy organs by 120 h p.i. The biodistribution data from 120 h p.i. reinforce this observation. [^89^Zr]Zr-DFO-^N297A^5B1 and [^89^Zr]Zr-DFO-5B1 yield comparable uptake values in tumor tissue (23.3 ± 22.5 and 16.21 ± 13.0 %ID/g, respectively) and exhibit similarly low accretion levels in healthy non-target organs such as the spleen (3.2 ± 0.7 and 5.1 ± 0.6 %ID/g), liver (4.1 ± 1.2 and 3.3 ± 2.6 %ID/g), and bone (2.6 ± 3.1 and 4.6 ± 1.7 %ID/g) (Figure 5).

## Discussion

While the focus of this investigation was undeniably the in vivo experiments, the in vitro assays nonetheless reinforce several key phenomena related to the interaction of radioimmunoconjugates with FcR. To wit, the binding of the immunoconjugates to FcRn is independent of glycosylation state, but the absence of the heavy chain glycans dramatically attenuates the affinity of the immunoconjugates for FcγRI and the affinity of their immunocomplexes for FcγRIIA, FcγRIIB, FcγRIIIA, and FcγRIIIB. Furthermore, the bioconjugation of DFO to glycosylated and aglycosylated mAb does not appreciably affect their binding (or that of their immunocomplexes) to FcγR.

**Figure 6:**
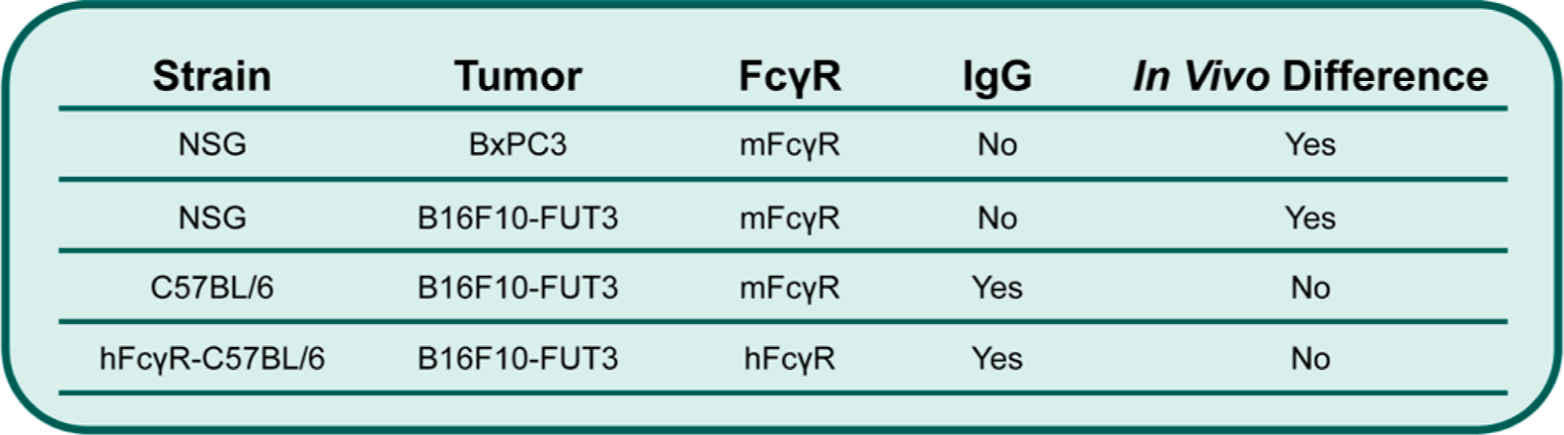
Summary of the in vivo results described herein.

Moving on to the in vivo work, the PET imaging and biodistribution experiments bring an explanation for the pharmacokinetic differences between glycosylated and aglycosylated radioimmunoconjugates into relief (Figure 6). We observed significant differences in the biodistributions of [^89^Zr]Zr-DFO-^N297A^5B1 and [^89^Zr]Zr-DFO-5B1 in NSG mice bearing either BxPC3 human PDAC xenografts or B16F10-FUT3 murine melanoma xenografts, with the aglycosylated radioimmunoconjugate producing higher tumoral uptake and lower healthy organ accretion in both cases. This phenomenon disappeared upon the switch to immunocompetent C57BL/6 and FcγR-humanized C57BL/6 mice. While these two mouse models express different FcγR—mFcγR for the former and hFcγR for the latter—both are differentiated from NSG mice by the presence of a functional immune system and, as a result, the production of endogenous IgG. More specifically, NSG mice have undetectable levels of endogenous IgG, while C57BL/6 and FcγR-humanized C57BL/6 mice have titers of ∼6–8 mg/mL.^32−34^ Accordingly, we hypothesize that the presence or absence of endogenous IgG lies at the core of our observations about the performance of glycosylated and aglycosylated immunoPET probes. In both types of C57BL/6 mice, the FcγR (whether murine or human) are occupied by endogenous IgG and thus cannot interfere with the behavior of the glycosylated radioimmunoconjugates, freeing them to travel throughout the body in a manner identical to that of their aglycosylated counterparts. In the NSG mice, however, the absence of IgG means that the mFcγR are unoccupied. As a result, these unoccupied mFcγR are free to bind glycosylated radioimmunoconjugates and influence their pharmacokinetics but are unable to affect their aglycosylated counterparts.

These data are consistent with our previous findings in this area. For example, there are dramatic differences in the behavior of glycosylated and deglycosylated variants of [^89^Zr]Zr-DFO-pertuzumab in tumor-bearing NSG mice that express both hFcγR and mFcγR but lack endogenous IgG. Significant disparities between the behavior of these two probes were not seen in tumor-bearing athymic nude mice that express mFcγR and are immunocompromised but nonetheless produce endogenous IgG. Sharma et al. noted that glycosylated ^89^Zr-labeled radioimmunoconjugates produced higher uptake in the healthy tissues of immunocompromised mice with low levels of endogenous IgG expression (i.e., SCID, NOD SCID, and NSG) compared to immunocompromised mice with endogenous IgG (i.e., athymic mice). Furthermore, this team noted that the suboptimal performance of glycosylated radioimmunoconjugates in NSG mice could be “rescued” via the use of deglycosylated or “Fc-silent” radioimmunoconjugates or blocking mFcγR with an excess of exogenous isotype control IgG.^18^ Curiously, Mangeat et al. did observe some differences in the biodistribution of a wildtype ^89^Zr-labeled radioimmunoconjugate and an analogue bearing a triple mutation meant to abrogate mFcγR binding (i.e., L234A/L235A/P329G) in tumor-bearing athymic nude mice. These changes were admittedly modest, however, and may thus stem from the fact that the LALAPG mutation is even more effective at abrogating FcγR interactions than aglycosylation or deglycosylation.^17^

Going forward, this work could have far-reaching implications in preclinical research. NSG, huNSG, and other highly immunocompromised mice without endogenous antibodies (e.g., SCID, NOD SCID, etc.) are common platforms for the evaluation of radioimmunoconjugates and antibody-drug conjugates, and the interactions between the unoccupied FcγR of these mice and these immunoconjugates could provide an unrealistic depiction of how these immunoglobulins will behave in more realistic immunocompetent mouse models and, eventually, patients. It will be important, however, to exercise caution when extending lessons from this work to the study of therapeutic mAb that also rely upon FcγR interactions for their efficacy; in these cases, the dual roles of FcγR in mediating pharmacokinetics and cytotoxicity surely make things much more complicated. Returning to nuclear medicine, it seems unlikely that deglycosylated or aglycosylated radioimmunoconjugates will dramatically outperform their fully glycosylated cousins in most patients, as the presence of endogenous IgG (and thus the occupation of hFcγR) will likely prevent the phenomenon that we have seen. That said, it is possible that “FcγR-silent” radioimmunoconjugates could confer advantages in a subset of more immunocompromised patients with lower serum IgG titers and thus higher concentrations of unoccupied FcγR.

## Conclusions

We believe that this investigation’s use of a genetically engineered, immunocompetent murine model with fully human FcγR makes it the most rigorous exploration to date of the interplay between radioimmunoconjugates and FcγR. We have clearly shown that aglycosylated ^89^Zr-labeled mAb outperform fully glycosylated analogues in tumor-bearing, immunocompromised NSG mice. This disparity, however, does not arise in immunocompetent C57BL/6 or FcγR-humanized C57BL/6 mice. Taken together, these data support the hypothesis that the absence of endogenous IgG in immunocompromised mice—and the consequent unoccupied state of their FcγR—lies at the heart of the improved performance of deglycosylated, aglycosylated, and “FcγR-silent” radioimmunoconjugates in these model systems. Ultimately, while the interaction between mAb-based probes and unoccupied FcγR is likely to have important implications for preclinical research, the impact of this work in the clinic remains to be seen and may only arise in highly immunocompromised patients with very low serum IgG titers.

## Supporting Information Available

MALDI-ToF spectrograms, radiochromatograms, radioimmunoconjugate stability assay data, and tables of biodistribution data (PDF)

## Abbreviations

FcR: Fc receptor
FcγR: Fcγ receptor
PET: positron emission tomography
mAb: monoclonal antibody
DFO: desferrioxamine
*p*-SCN-DFO: 1-(4-isothiocyanatophenyl)-3-[6,17-dihydroxy-7,10,18,21-tetraoxo-27-(*N*-acetylhydroxylamino)-6,11,17,22-tetraazaheptaeicosine] thiourea
CT: computed tomography
PBS: phosphate-buffered saline
MWCO: molecular weight cutoff
EDTA: ethylenediaminetetraacetic acid
iTLC: instant thin layer chromatography
hIgG: antihuman immunoglobulin G antibody
%ID/g: percentage injected dose per gram of tissue
ELISA: enzyme-linked immunosorbent assay
MSKCC: Memorial Sloan Kettering Cancer Center
SE-HPLC: size exclusion high-performance liquid chromatography
IACUC: institutional animal care and use committee.
