# Supplementary material for: Exploring the Interplay Between Radioimmunoconjugates and Fcγ Receptors in Genetically Engineered Mouse Models of Cancer
Source: ACS Pharmacol Transl Sci. 2024 Oct 17;7(11):3452–61. doi: 10.1021/acsptsci.4c00275 (PMC11555515; doi:10.1021/acsptsci.4c00275)
Supplement: Supplementary file 1 — pt4c00275_si_001.pdf [file pt4c00275_si_001.pdf]

Supporting Information

Exploring the Interplay between Radioimmunoconjugates and Fcγ Receptors in Genetically Engineered Mouse Models of Cancer

Cindy Rodriguez[1,2,3], Samantha M. Sarrett[1,2,4], Joni Sebastiano[1,2,4], Samantha Delaney[1,2,4], Shane McGlone[1], Meena M. Hosny[1], Sarah Thau[1], Stylianos Bournazos[5], Brian M. Zeglis[1,2,3,4,6]

[1] Department of Chemistry, Hunter College, City University of New York, New York, New York

[2]Department of Radiology, Memorial Sloan Kettering Cancer Center, New York, New York

[3]Ph.D. Program in Chemistry, Graduate Center of City University of New York, New York, New York

[4]Ph.D. Program in Biochemistry, Graduate Center of City University of New York, New York, New York

[5]Laboratory of Molecular Genetics and Immunology, The Rockefeller University, 1230 York Avenue, New York, NY, 10065, USA

[6]Department of Radiology, Weill Cornell Medical College, New York, New York

Corresponding Author: Brian M. Zeglis: 413 East 69th Street, New York, NY, 10021; Phone: 212-896-0433; E-mail: bz102@hunter.cuny.edu

Keywords: Molecular imaging, radioimmunoconjugate, positron emission tomography, PET, single photon emission computed tomography, SPECT, radioimmunotherapy, radiopharmaceutical therapy, immunotherapy, immune system, Fc receptor, Fcγ receptors, heavy chain glycans, aglycosylated antibody.

SUPPLEMENTAL FIGURES

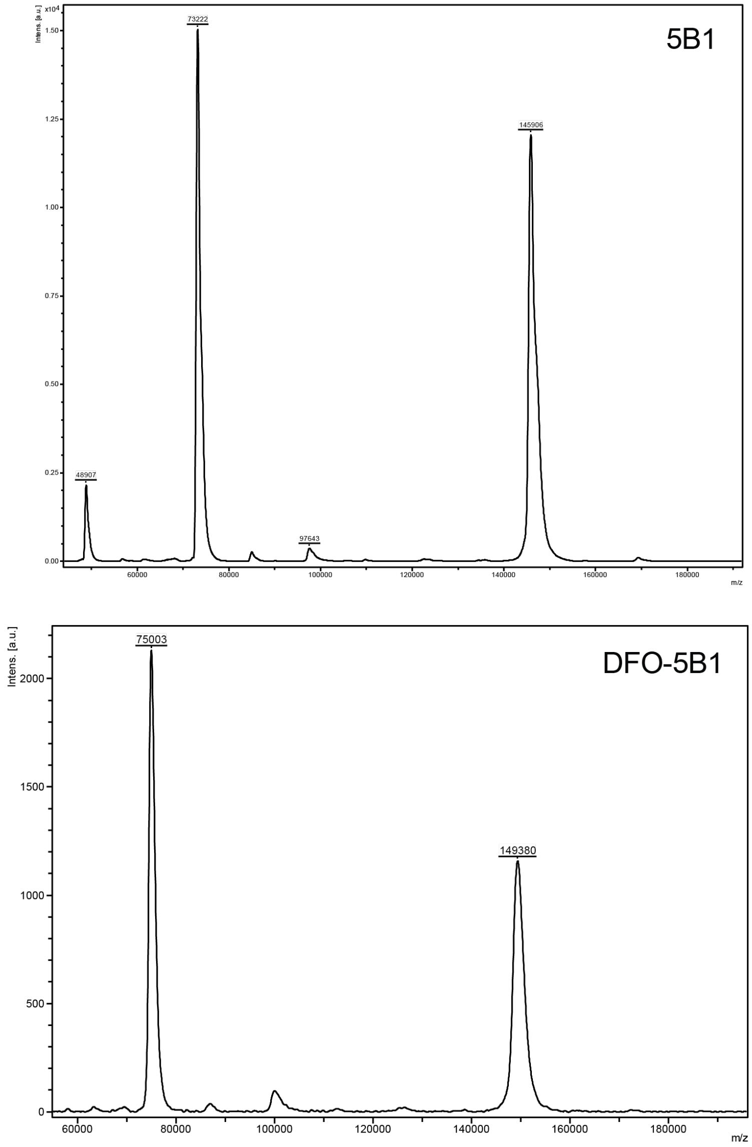

Figure S1. Representative MALDI-ToF spectrograms used to determine the chelator-to-antibody ratio of DFO-5B1.

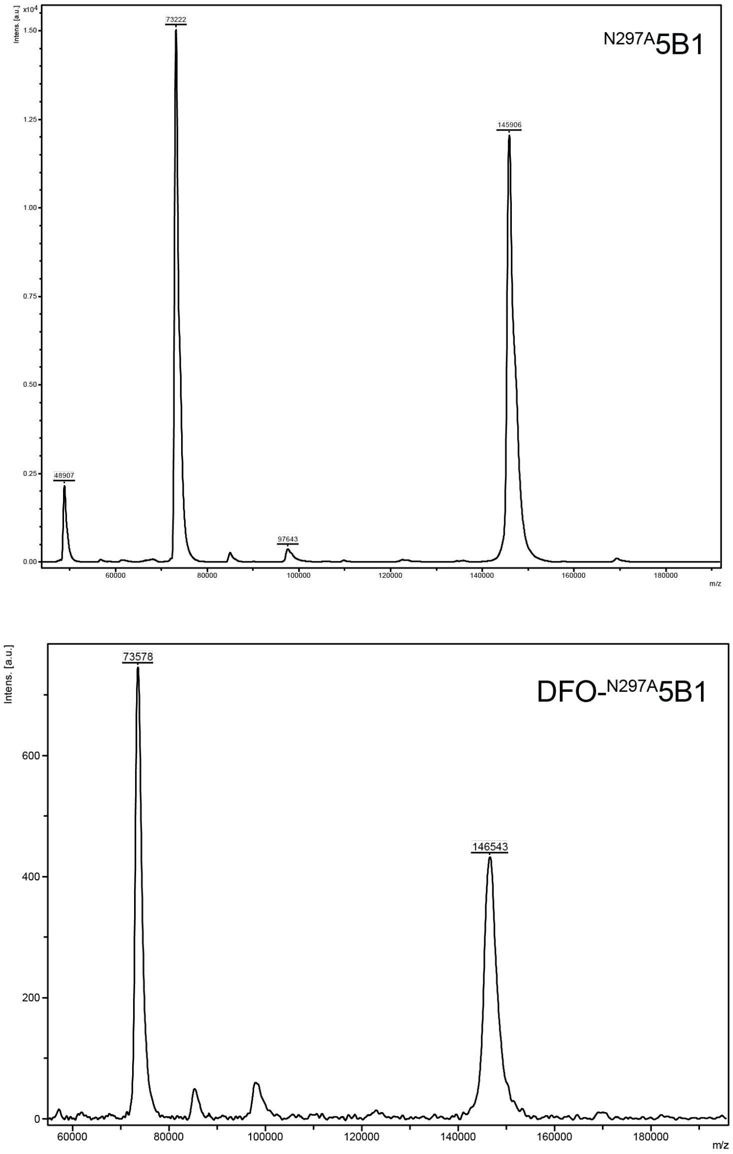

Figure S2. Representative MALDI-ToF spectrograms used to determine the chelator-to-antibody of DFO-^{N297A}5B1.

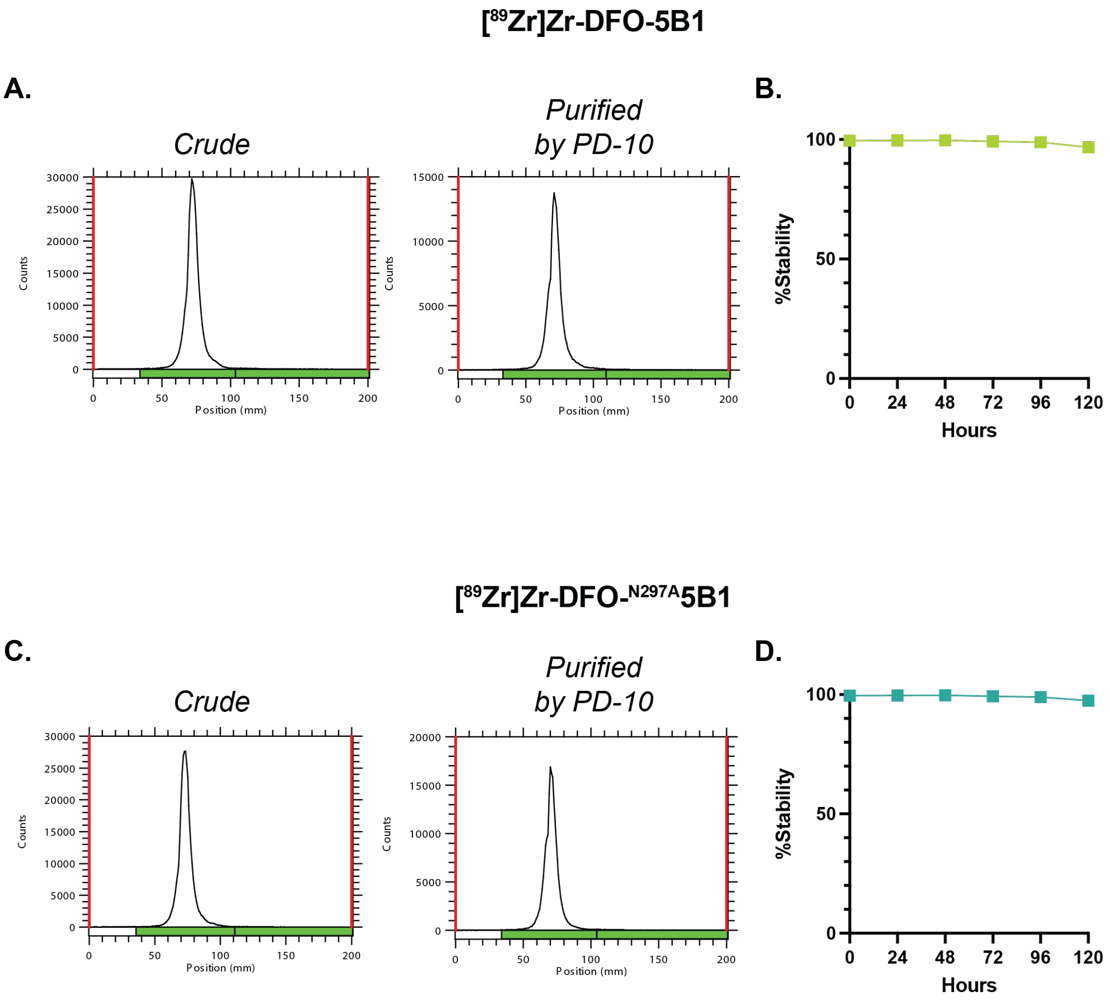

Figure S2. (A and C) Representative radio-iTLC chromatogram of (A) [^{89}Zr]Zr-DFO-5B1 and (C) [^{89}Zr]Zr-DFO-^{N297A}5B1before (left) and after (right) purification obtained using an eluent of 50 mM EDTA (pH 5.0). The peak at ~75 mm corresponds to the radiolabeled antibody, while any [^{89}Zr]Zr-EDTA would be seen at the solvent front (*i.e.* ~150 mm). Both constructs exhibited >98% radiochemical purity both before and after purification via gel filtration, and all radiolabeling studies and iTLC scans were performed in triplicate. (B and D) Human serum stability data for [^{89}Zr]Zr-DFO-5B1 (B) and [^{89}Zr]Zr-DFO-^{N297A}5B1 (D). After purification, each radioimmunoconjugate was incubated in 0.5 mL human serum on a thermomixer at 37 °C and 400 rpm. After 0, 24, 48, 72, 96, and 120 h, each radioimmunoconjugate was assayed using a radio-iTLC scanner with 50 mM EDTA (pH 5.0) as an eluent. The % of radioactivity remaining with the immunoconjugate at each timepoint was plotted using GraphPad Prism 8.0 (n = 3).

SUPPLEMENTAL TABLES

Table 1. Biodistribution data collected 120 h after the intravenous administration of [^{89}Zr]Zr-DFO-5B1 or [^{89}Zr]Zr-DFO-^{N297A}5B1 [3.7–3.9 MBq (100–105 μCi), 20–21 μg in 100 μL of PBS] to NSG mice bearing subcutaneous CA19-9-expressing BxPC3 PDAC xenografts (n = 4). Values are in units of %ID/g and are expressed as mean ± standard deviation.

Tissue	[^{89}Zr]Zr-DFO-5B1	[^{89}Zr]Zr-DFO-^{N297A}5B1
**Blood**	0.04 ± 0.0	6.3 ± 1.4
**Tumor**	8.3 ± 6.4	73.9 ± 22.3
**Heart**	1.0 ± 0.0	1.8 ± 0.9
**Lungs**	0.8 ± 0.6	4.5 ± 0.8
**Liver**	5.7 ± 3.4	3.9 ± 2.1
**Spleen**	21.8 ± 4.5	3.4 ± 1.0
**Pancreas**	0.2 ± 0.0	0.4 ± 0.4
**Stomach**	0.4 ± 0.1	0.3 ± 0.1
**S. Intestine**	1.9 ± 0.3	0.7 ± 0.1
**L. Intestine**	0.5 ± 0.2	0.9 ± 0.4
**Kidneys**	1.0 ± 0.3	3.3 ± 1.1
**Muscle**	0.1 ± 0.0	0.2 ± 0.1
**Bone**	7.5 ± 1.8	2.5 ± 0.4
**Skin**	1.2 ± 0.2	3.5 ± 0.6

Table 2. Biodistribution data collected 120 h after the intravenous administration of [^{89}Zr]Zr-DFO-5B1 or [^{89}Zr]Zr-DFO-^{N297A}5B1 [3.7–3.9 MBq (100–105 μCi), 20–21 μg in 100 μL of PBS] to NSG mice bearing subcutaneous CA19-9-expressing B16F10-FUT3 xenografts (n = 4).Values are in units of %ID/g and are expressed as mean ± standard deviation.

Tissue	[^{89}Zr]Zr-DFO-5B1	[^{89}Zr]Zr-DFO-^{N297A}5B1
**Blood**	0.2 ± 0.1	2.3 ± 0.8
**Tumor**	3.7 ± 1.2	7.9 ± 3.1
**Heart**	0.9 ± 0.3	0.9 ± 0.1
**Lungs**	0.7 ± 0.7	0.8 ± 0.4
**Liver**	6.2 ± 3.0	5.0 ± 0.3
**Spleen**	27.5 ± 11.1	1.6 ± 0.4
**Pancreas**	0.2 ± 0.0	0.2 ± 0.1
**Stomach**	0.3 ± 0.2	0.2 ± 0.1
**S. Intestine**	1.9 ± 0.5	0.3 ± 0.1
**L. Intestine**	0.6 ± 0.4	0.3 ± 0.1
**Kidneys**	1.0 ± 0.4	3.6 ± 2.0
**Muscle**	0.2 ± 0.1	0.1 ± 0.0
**Bone**	7.3 ± 1.8	1.9 ± 0.3
**Skin**	1.3 ± 0.2	1.4 ± 0.3

Table 3. Biodistribution data collected 120 h after the intravenous administration of [^{89}Zr]Zr-DFO-5B1 or [^{89}Zr]Zr-DFO-^{N297A}5B1 [3.7–3.9 MBq (100–105 μCi), 20–21 μg in 100 μL of PBS] to C57BL/6 mice bearing subcutaneous CA19-9-expressing B16F10-FUT3 xenografts (n = 4).Values are in units of %ID/g and are expressed as mean ± standard deviation.

Tissue	[^{89}Zr]Zr-DFO-5B1	[^{89}Zr]Zr-DFO-^{N297A}5B1
**Blood**	9.1 ± 3.0	8.3 ± 1.5
**Tumor**	20.7 ± 14.0	15.7 ± 8.0
**Heart**	1.9 ± 1.5	2.4 ± 0.6
**Lungs**	1.4 ± 0.6	3.2 ± 1.4
**Liver**	1.7 ± 0.3	6.0 ± 2.5
**Spleen**	3.8 ± 1.7	2.9 ± 1.6
**Pancreas**	0.5 ± 0.2	0.5 ± 0.2
**Stomach**	1.0 ± 0.5	1.0 ± 0.4
**S. Intestine**	1.4 ± 0.4	1.0 ± 0.3
**L. Intestine**	0.4 ± 0.1	0.8 ± 0.3
**Kidneys**	2.8 ± 0.6	5.2 ± 1.3
**Muscle**	0.7 ± 0.8	0.3 ± 0.1
**Bone**	3.9 ± 0.8	2.2 ± 0.7
**Skin**	4.1 ± 1.4	1.8 ± 1.0

Table 4. Biodistribution data collected 120 h after the intravenous administration of [^{89}Zr]Zr-DFO-5B1 or [^{89}Zr]Zr-DFO-^{N297A}5B1 [3.7–3.9 MBq (100–105 μCi), 20–21 μg in 100 μL of PBS] to FcγR-humanized C57BL/6 mice bearing subcutaneous CA19-9-expressing B16F10-FUT3 xenografts (n = 4).Values are in units of %ID/g and are expressed as mean ± standard deviation.

Tissues	[^{89}Zr]Zr-DFO-5B1	[^{89}Zr]Zr-DFO-^{N297A}5B1
**Blood**	15.6 ± 7.1	10.1 ± 3.4
**Tumor**	16.2 ± 13.0	23.3 ± 22.5
**Heart**	5.0 ± 5.3	4.6 ± 1.5
**Lungs**	5.0 ± 3.4	4.5 ± 4.2
**Liver**	3.3 ± 2.6	4.1 ± 1.2
**Spleen**	5.1 ± 0.6	3.2 ± 0.7
**Pancreas**	2.2 ± 1.0	1.1 ± 0.8
**Stomach**	1.0 ± 0.4	1.3 ± 0.7
**S. Intestine**	0.9 ± 0.4	1.3 ± 0.7
**L. Intestine**	0.8 ± 0.4	0.8 ± 0.5
**Kidneys**	3.3 ± 1.1	5.7 ± 3.1
**Muscle**	0.8 ± 0.2	0.9 ± 0.4
**Bone**	4.6 ± 1.7	2.6 ± 3.1
**Skin**	6.5 ± 2.9	9.8 ± 8.1
